# Large fluctuations in the effective population size of the malaria mosquito *Anopheles gambiae s.s*. during vector control cycle

**DOI:** 10.1111/eva.12094

**Published:** 2013-08-07

**Authors:** Theresa K Hodges, Giridhar Athrey, Kevin C Deitz, Hans J Overgaard, Abrahan Matias, Adalgisa Caccone, Michel A Slotman

**Affiliations:** 1 Department of Entomology, Texas A&M UniversityCollege Station, TX, USA; 2 Department of Mathematical Sciences and Technology, Norwegian University of Life SciencesÅs, Norway; 3 Medical Care Development International Inc. Malabo, Equatorial GuineaNew Haven, CT, USA; 4 Department of Ecology and Evolutionary Biology, Yale UniversityNew Haven, CT, USA

**Keywords:** *Anopheles gambiae*, approximate Bayesian computation, effective population size, indoor residual spraying, malaria, vector control

## Abstract

On Bioko Island, Equatorial Guinea, indoor residual spraying (IRS) has been part of the Bioko Island Malaria Control Project since early 2004. Despite success in reducing childhood infections, areas of high transmission remain on the island. We therefore examined fluctuations in the effective population size (*N*_*e*_) of the malaria vector *Anopheles gambiae* in an area of persistent high transmission over two spray rounds. We analyzed data for 13 microsatellite loci from 791 *An. gambiae* specimens collected at six time points in 2009 and 2010 and reconstructed the demographic history of the population during this period using approximate Bayesian computation (ABC). Our analysis shows that IRS rounds have a large impact on *N*_*e*_, reducing it by 65%–92% from prespray round *N*_*e*_. More importantly, our analysis shows that after 3–5 months, the *An. gambiae* population rebounded by 2818% compared shortly following the spray round. Our study underscores the importance of adequate spray round frequency to provide continuous suppression of mosquito populations and that increased spray round frequency should substantially improve the efficacy of IRS campaigns. It also demonstrates the ability of ABC to reconstruct a detailed demographic history across only a few tens of generations in a large population.

## Introduction

Malaria is a major public health threat throughout large areas of the world. In 2010, malaria cases numbered approximately 216 million, resulting in more than 655 000 deaths. Of these, 86% occurred in children less than 5 years of age, and approximately 90% of all deaths occur in sub-Saharan Africa (World Malaria Report 2011). In Africa, transmission is largely accomplished by a few highly efficient vectors, of which *Anopheles gambiae s.s*. is the most important. The high vectorial capacity of this species is due to its wide distribution, close association with human habitat, high preference for feeding on humans, and tendency to readily enter houses at night to feed. *An. gambiae* belongs to a complex of seven morphologically cryptic species (Davidson 1962; White 1974; Hunt et al. 1998), several of which are also important vectors in various regions of sub-Saharan Africa. *An. gambiae s.s*. is further subdivided into the M and S molecular forms, which are widely recognized to be two incipient species, although separate species status was recently proposed for *An. gambiae* (M) (syn. *A. coluzzii*) (Coetzee et al. 2013).

In the recent decade, antivector interventions have increased dramatically as part of malaria control initiatives in many countries in sub-Saharan Africa. This typically includes the large-scale distribution of insecticide-treated bed nets (ITNs), recently supplanted by long-lasting insecticidal nets (LLINs), and/or indoor residual spraying (IRS). Between 2006 and 2008, close to 290 million ITNs/LLINs were distributed in sub-Saharan Africa, covering approximately 578 million people, whereas IRS programs covered about 81 million people (World Malaria Report 2011). IRS involves the periodic spraying of the inside walls of houses with an insecticide with a long-lasting residual effect. This targets malaria vectors such as *An. gambiae s.s*. that have a strong tendency to feed and rest indoors. These methods have been successful in reducing malaria morbidity and mortality in various locations (Kleinschmidt et al. 2009; Zhou et al. 2010; Okumu and Moore 2011), have frequently led to a decrease in mosquito abundance (Takken 2002; Sharp et al. 2007; Bayoh et al. 2010), and on occasion have even eliminated vectors from controlled areas (Pringle 1967; Sharp et al. 2007).

**Figure 1:**
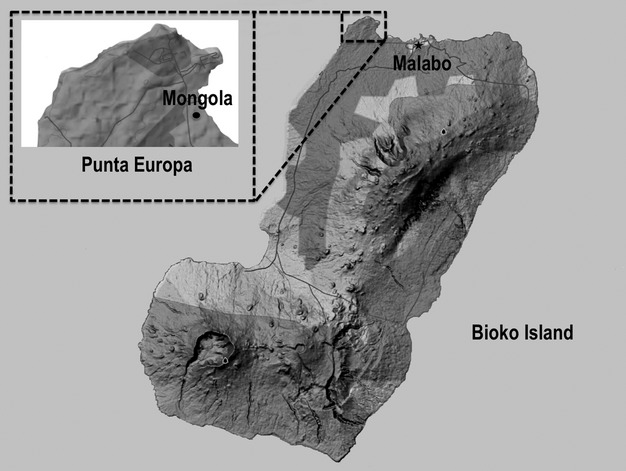
A map of Bioko Island indicating the location of our sampling site, Mongola, within the Punta Europa area.

On Bioko Island, Equatorial Guinea, vector control is implemented under the Bioko Island Malaria Control Project (BIMCP) and is based on biannual IRS and a single mass LLIN distribution round in late 2007/early 2008. The BIMCP also includes case management, preventive treatment during pregnancy, monitoring and evaluation, a public information campaign, and operational research. It has resulted in a remarkable 57% drop in child mortality (Kleinschmidt et al. 2009) and eliminated two vectors from the island: *An. gambiae* (S) and *Anopheles funestus* (Sharp et al. 2007; Overgaard et al. 2012). Despite these successes, *An. gambiae* (M) and *Anopheles melas* are still important vectors on the island, and localized areas with high transmission and human parasitemia levels remain. One of these areas is Punta Europa in the northwest corner of the island (Fig. 1), which is home to several small villages and the operational base of foreign oil companies.

Although vector control has in many instances reduced vector abundance, this is not always the case (Takken 2002), and quantifying changes in mosquito population size can be difficult to achieve because of various limitations of collection methods. For example, mosquito collections can be greatly affected by the weather at the time of collections, during indoor collections, mosquitoes may be repelled by insecticides used in IRS or ITNs, and human volunteers may vary greatly in their attractiveness to mosquitoes during human landing catches (Silver 2008). More reliable direct methods such as mark–release–recapture (MRR) have been used to estimate the size of mosquito populations and their dispersal [e.g., (Costantini et al. 1996; Touré et al. 1998; Silver 2008)]. However, MRR can be logistically challenging, and it has not been used to study the impact of vector control on malaria mosquitoes. A few studies have attempted to examine the impact of vector control on malaria mosquito populations using indirect genetic methods by either estimating changes in the effective population size (*N*_*e*_) [e.g., (Wondji et al. 2005; Athrey et al. 2012b)] or by looking for a genetic signature of a population bottleneck [e.g., (Pinto et al. 2002, 2003)].

*N*_*e*_, which is defined as the size of an ideal population that is subject to genetic drift at the same rate as the actual population (Wright 1931, 1938), can be notoriously difficult to measure accurately in natural populations. Nonetheless, a wide variety of *N*_*e*_ estimators are available, each with its own advantages and disadvantages (reviewed in Luikart et al. 2010). Three types of estimators that have been widely used are the recently developed sibship method implemented in Colony (Wang 2009), the linkage disequilibrium method implemented in LDN_*e*_ (Waples and Do 2008), and the temporal method implemented in MLN_*e*_ (Wang and Whitlock 2003). These estimators are most precise for smaller, isolated populations, and may not be very informative about mosquito populations, which are typically large.

Wondji et al. (2005) examined the impact of vector control on the *N*_*e*_ of the malaria vector *Anopheles arabiensis*, a sibling species of *An. gambiae*, in a village in northern Cameroon. They used a temporal method to estimate *N*_*e*_ before and after an ITN distribution campaign. This study detected a measurable, though nonsignificant, decline in *N*_*e*_ following ITN distribution. However, the decline was transient, which the authors attributed to the small scale of the intervention and the migration of *An. arabiensis* mosquitoes from neighboring populations into the study village.

Pinto et al. (2002, 2003) studied the effect of DDT-based IRS on *An. gambiae* populations in the archipelago of São Tomé and Principe. Although indoor mosquito densities were significantly reduced during this campaign (Ceita 1986), no evidence of a bottleneck was detected in *An. gambiae* populations on the two islands (Pinto et al. 2002, 2003). The authors therefore called into question the effectiveness of IRS in reducing malaria vector populations and proposed that exophagic and exophilic tendencies of the vector on the island of São Tomé (Sousa et al. 2001) might have prevented exposure of the vector to the insecticide. However, it was shown more recently that the bottleneck analyses (Cornuet and Luikart 1996) used by Pinto et al. (2002, 2003) can produce false-negative results if the bottleneck occurred less than 2*N*_*e*_ to 4*N*_*e*_ generations ago (Cristescu et al. 2010). Therefore, the lack of a bottleneck signal in these *An. gambiae* populations is not conclusive evidence for a lack of impact of vector control on *N*_*e*_.

More recently, approximate Bayesian computation (ABC) (Beaumont et al. 2002) has been used to estimate *N*_*e*_ (e.g., Wegmann and Excoffier 2010; Lombaert et al. 2011; Athrey et al. 2012a). ABC is a coalescent simulation-based approach that allows a comparison between competing demographic scenarios. Briefly, demographic scenarios of interest are defined and used to simulate a large number of data sets whose characteristics are similar to a real data set (i.e., number and type of loci, number of individuals). A number of population genetic summary statistics are then used to compare the simulated data sets to the actual data set to derive posterior probabilities for each of the competing scenarios. Once the most likely scenario is identified, its simulated data sets are used to construct posterior density distributions of the parameters of interest (e.g., *N*_*e*_ and the timing of changes in *N*_*e*_). This approach reduces bias and increases precision (Cornuet et al. 2008, 2010; Luikart et al. 2010).

In a previous study, we used ABC analyses to provide the first conclusive evidence that IRS and LLINs can have a large impact on the effective size of malaria mosquito populations (Athrey et al. 2012b). We showed that *N*_*e*_ was reduced between 55% and 87% in six of the seven study populations in Equatorial Guinea, coincidental with the start of the vector control (IRS and LLINs). The *An. gambiae* M population from Mongola, Bioko Island, which is the subject of the present study, was reduced by 79% around the start of the vector control campaign.

However, our previous study was not designed to examine fluctuations in *N*_*e*_ associated with individual IRS rounds. In the BIMCP, spray rounds were conducted every 6 months, and the residual efficacy of the carbamate insecticide has been a matter of concern. Although the IRS campaign on Bioko Island did substantially reduce child mortality (Kleinschmidt et al. 2009), Bradley et al. (2012) recently reported that the efficacy of the insecticide begins to decline 4 months after spraying, killing 73% (2010 data) or 81% (2011 data) of mosquitoes exposed to sprayed walls in WHO cone bioassays, as compared to 100% soon after spraying. This was correlated with an increase in human parasitemia rates from approximately 18% to 28% between 3 and 5 months after spraying. Additionally, a recent study in Benin showed that carbamate-based IRS carried out every 8 months provides no additional parasitemia reduction, when used in addition to LLINs (Corbel et al. 2012).

The dynamics of mosquito populations during an IRS campaign is an important issue as it pertains to the ability of mosquito populations to rebound between spray rounds, when the efficacy of the insecticide may drop below effective levels. Therefore, we took advantage of the availability of a time series of six *An. gambiae* M samples from 2009 to 2010 from Mongola in the Punta Europa area of Bioko Island and conducted an ABC analysis to assess patterns and levels of *N*_*e*_ fluctuations using 13 microsatellite and 791 specimens. In this area, entomological inoculation rates and human parasitemia rates remained high despite 5 years of continued IRS (Bradley et al. 2012; Overgaard et al. 2012). Our data provide important information on the dynamics of *An. gambiae* M populations during an IRS campaign by showing that IRS rounds dramatically reduce *N*_*e*_, but that *N*_*e*_ recovers between 3 and 5 months following the spraying of insecticides. Our results imply that increasing the frequency of IRS rounds will have a dramatic impact on malaria transmission.

Because ABC has become a powerful approach to explore questions about demographic histories in diverse fields such as population genetics, phylogeography, and epidemiology in recent years (Beaumont 2010), we used ABC as our main approach to detect fluctuations in *N_e_* over the timescale of our study period. ABC was able to detect a pattern of multiple contractions and expansions in a large population over only about 30 generations. Our analyses of the complex demographic history of *An. gambiae* from the Punta Europa area highlight the ability of ABC to reconstruct demographic histories in large populations over contemporary timescales.

## Materials and methods

### Study location

This study was conducted in Mongola (3°45.88788′N, 8°43.30314′E) in the Punta Europa area of Bioko Island, Equatorial Guinea (Fig. 1). Mongola is a small, rural village located outside of the capital city of Malabo and is close to two other small villages, Biabia and Cacahual. The Punta Europa area experiences a wet season from April through October and a dry season from November through March. Rainfall data for March 2009 through November 2009 were collected in the adjacent village of Cacahual, and total rainfall for March–November 2009 was 1919 mm. Average monthly rainfall from 2004 to 2007 was used for months for which no 2009 rain data were available (Overgaard et al. 2012).

### Vector control

Antivector interventions in the form of IRS started on Bioko Island in 2004 as part of the Bioko Island Malaria Control Project (BIMCP), in cooperation with the National Malaria Control Program within the Equatoguinean Ministry of Health and Social Welfare. This was implemented by Medical Care Development International with support from a private donor consortium led by Marathon Oil Corporation and the government of Equatorial Guinea. At least 80% of houses on Bioko Island were initially sprayed in 2004 with pyrethroid class insecticide Deltamethrin™ (Bayer CropScience, Isando, South Africa). In 2005, these insecticides were replaced with Ficam™ (bendiocarb; Bayer CropScience), a carbamate insecticide, after high levels of *kdr* (L1014F), a target site mutation conferring resistance to pyrethroids and DDT, were detected in mosquitoes during the course of routine monitoring (Kleinschmidt et al. 2005; Sharp et al. 2005). This carbamate insecticide was used in all subsequent spray rounds. In late 2007 and early 2008, deltamethrin-treated LLINs were distributed to residents on Bioko Island, including Mongola, through a single mass campaign. These nets can be effective for 3 years, but by 2009, only 27% of children <15 years were sleeping under a net, and this number dropped to around 14% by 2010 (I. Kleinschmidt, personal communication).

Prior to our collections, an IRS round was conducted in Mongola from September 29, 2008 to October 2, 2008. Two additional spray rounds were conducted during our study period. The first of these occurred in June 2009 during the wet season, followed by a second spray round in December 2009 during the dry season (Table 1).

**Table 1:** Sample collection days, collection method, sample sizes, and dates of the two IRS rounds prior and during the sampling period

Year	Mosquito collection dates	Collection method*	Sample size (*N*)	IRS dates
2008				September 29–October 2
2009	March 23–March 27	HLC, LTC	125	
	May 17–May 19	HLC	142	
				June 12–June 16
	July 6–July 8	HLC	137	
	August 30–September 1	HLC, LTC	147	
	November 9–November 11	HLC	146	
				December 18–December 19
2010	April 12–April 13	HLC, LTC	95	

* HLC refers to human landing catches, and LTC stands for light trap catches.

### Mosquito sampling

Adult female anopheline mosquitoes were collected in Mongola using human landing catches (HLCs) and CDC light trap collections (LTCs) in March, May, July, September, and November 2009, as part of the entomological monitoring component of the BIMCP. HLCs were conducted both indoors and outdoors, whereas LTCs were all conducted indoors. In these samples, *An. gambiae s.s*. (M) was the dominant vector (99.7%; Overgaard et al. 2012). From these collections, 950 *An. gambiae* (M) mosquitoes were included in our study (Table 1). In addition, microsatellite data from Athrey et al. (2012b) for 95 samples collected from the same location in April 2010 were also included.

### DNA extraction and genotyping of microsatellites

DNA from heads and thoraxes of female mosquitoes preserved in 70% ethanol was extracted on a Qiagen Biosprint (Qiagen Inc, Valencia, CA, USA). Extracted DNA was resuspended in 200 μL elution buffer and stored at −20°C. Species diagnostics were performed following Fanello et al. (2002). A total of 697 samples were genotyped at 13 species-specific autosomal microsatellite loci: *AG2H79*, *AG2H325*, *AG2H603*, *AG2H720*, *AG2H770*, *AG2H787*, *AG3H59*, *AG3H93*, *AG3H119*, *AG3H242*, *AG3H249*, *AG3H577*, *and AG3H817* (Zheng et al. 1996). Microsatellite PCRs were carried out in 20-μL reactions with 10 mm dNTP, 2 mm MgCl_2_, 1 μmol fluorescently labeled (6-FAM, NED, or HEX) forward primer and 1 μmol reverse primer, in a Promega® reagent master mix with GoTaq Flexi Polymerase (Promega Corp., Madison, WI, USA) and 5× buffer. PCR products were run on an ABI 3730x at the DNA Analysis Facility on Science Hill (Yale University, New Haven, CT, USA).

### Data analysis

Genotype calls were performed in GeneMarker (Softgenetics, State College, PA, USA). Genotype data files were formatted using GENALEX 6 (Peakall and Smouse 2006). If we were unable to obtain genotypes for two loci for a single mosquito, it was excluded. The final analyses were performed on a total of 791 individuals (Table 1). The data were examined for the presence of null alleles using the program Micro-Checker 2.2.3 (Van Oosterhout et al. 2004). Samples with possible null alleles were regenotyped, and the data were re-examined using Micro-Checker 2.2.3. The web program Genepop (Raymond and Rousset 1995; Rousset 2008) was used to test for the presence of linkage disequilibrium between loci. Unbiased expected heterozygosity (*H*_Exp_) was calculated for each temporal sample using GENALEX 6. Allelic richness (*A*_R_) for each temporal sample was calculated using FSTAT version 2.9.3 (Goudet 2002), and the means from all temporal samples were compared with an anova using the web-based tool provided by the Statistics Online Computational Resource ((Dinov 2006) http://www.socr.ucla.edu).

Approximate Bayesian computation (ABC) implemented in the DIYABC program (Cornuet et al. 2008, 2010) was used to examine *N*_*e*_ changes throughout 2009 and early 2010. ABC (Beaumont et al. 2002) is a coalescent-based method that identifies the scenario that best explains the data using summary statistics from observed population samples and comparing them with data simulated from scenarios describing alternative evolutionary histories. We tested a total of six demographic models (Table 2). These were designed to examine the impact of IRS rounds and rainfall on *N*_*e*_. Detailed descriptions of how the models were coded are presented in Table S1.

**Table 2:** The demographic models tested using approximate Bayesian computation on *Anopheles gambiae* (M-form) from Mongola, Bioko Island. A more detailed description of the demographic models is provided in Table S1

Scenario	Description
1	*N*_*e*_ is constant
2	*N*_*e*_ is reduced by IRS rounds, but starts to recover soon after
3	*N*_*e*_ is reduced by each subsequent IRS round and does not recover between rounds
4	*N*_*e*_ fluctuates based on a positive correlation with rainfall
5	*N*_*e*_ fluctuates based on a negative correlation with rainfall
6	*N*_*e*_ is reduced by IRS rounds and declines for 3 months before recovering by month 4

One million data sets were simulated for each scenario, and Euclidean distances between observed and simulated data sets were computed. As recommended, the top 1% of the simulated data sets most similar to the real data set were retained for each scenario and used in a logistic regression analyses to derive the posterior probabilities of each scenario (Beaumont et al. 2002). The general mutation model for microsatellites, which includes both stepwise and infinite-allele modes of mutation, was used. Mutation rates varied around the same median value of 6.34 × 10^−4^. We assumed 18 generations/year, but to examine the robustness of our results to variation in this parameter, we also compared the six demographic scenarios using 12 and 24 generations/year.

ABC analyses of the Mongola (Punta Europa) data set from Athrey et al. (2012b) that spans 6 years were used to test whether 12, 15, 18, 20, or 24 generations/year best represents reality. For this, we used the preferred bottleneck scenario and only varied the number of generations/year. This analysis showed that 18 generations/year was the best supported (posterior probability = 0.50), followed by 20 (posterior probability = 0.29) and 24 (posterior probability = 0.21) generations/year. Posterior probabilities for 12 and 15 generations/year were both zero.

Estimates of *N*_*e*_ and the timing of demographic events were based on the top 1% of the simulated data for the most likely scenario (Beaumont et al. 2002). We took the median of the posterior density distributions as our estimate. The 95% credibility intervals (Cr.I.) around each estimated parameter were obtained by excluding all values below 5% frequency from the posterior density distribution. These calculations were performed in RStudio, running the open source statistical platform R (R Core Team 2012).

To explore how much the results of our ABC analysis depend on each of eight summary statistics used to compare the competing scenarios, we conducted two sets of additional analyses in which the best-supported scenario was compared with either the second best scenario or the scenario representing a constant *N*_*e*_. During these analyses, each of the eight summary statistics was removed in turn.

In addition to ABC, three additional methods were used to estimate *N*_*e*_. We applied the sibship analysis implemented in the program Colony (Jones and Wang 2010), which uses the frequencies of full- and half-sibs in the population to derive estimates of *N*_*e*_ for each sample. For each estimate, we assumed a random mating system of female monogamy and male polygamy and incorporated an error rate of 0.015 for each locus. We selected the maximum-likelihood option and three runs of medium length in Colony for all *N*_*e*_ estimates.

The linkage disequilibrium method (Hill 1981) implemented in the program LDN_*e*_ (Waples and Do 2008) was also used to estimate *N*_*e*_ (Waples and Do 2008, 2010). This method is based on the presence of linkage disequilibrium between loci and provides point estimates of *N*_*e*_ in immediate past generations for each of the temporal samples. LDN_*e*_ includes a correction for a bias that occurs when alleles are present at low frequencies (Waples 2006). We ran LDN_*e*_ using a model of random mating and used the jackknife option to construct 95% confidence intervals. We report *N*_*e*_ estimates based on analyses that excluded rare alleles at frequencies <0.02 or <0.005. Removing these reduces the upward bias in *N*_*e*_ due to using loci with rare alleles (Waples and Do 2010).

Finally, *N*_*e*_ was also estimated using a maximum-likelihood approach as implemented in the program MLN_*e*_ (Wang 2001; Wang and Whitlock 2003). This method uses changes in allele frequencies between temporal samples to estimate *N*_*e*_, assuming that these are due to genetic drift. This analysis was performed on all pairings of the six temporal samples that were separated by at least 8–10 generations.

### *Post hoc* modeling

On June 22, 2009, an extraordinary amount (177 mm) of rainfall was deposited on Punta Europa in a period of 24 h. This event took place about 7–10 days following the first spray round during our study period. This deluge could potentially have caused large-scale larval mortality by washing out larval habitats. For example, Paaijmans et al. (2007) reported that larval mortality increased by up to 23% due to heavy rainfall. To examine the potential impact of such an event on *N*_*e*_, we constructed a demographic projection model.

This stage-based model with daily survival probabilities (From Olayemi and Ande 2009) was parameterized with a transition matrix, using a density-dependent logistic growth function (Jensen 1997), *N*_(*t *+ 1) _= *N*_*t*_ + ((*K *− *N*_*t*_)/*K*) × (*M*) × *N*_*t*_, where *N*_*t*_ is the population vector at time *t*, *N*_(*t *+ 1)_ is the population vector at time *t* + 1, *K* is the carrying capacity, and *M* is the age-based transition matrix. In this model, K for the adult class was defined as *N*_*e*_, with the proportional sizes for the other classes determined by the stable stage distribution. We modeled a 100-time step period (48 h/step) and imposed two perturbation events of different magnitude to simulate the potential impact of the deluge. The model was designed to predict the impact of these events on adult *N*_*e*_ in an *An. gambiae* population with a *N*_*e*_ of 5692 (= the estimated *N*_*e*_ at the time of the deluge according to ABC analyses). To be conservative regarding survival probabilities of larval stages, we modeled two extreme perturbation events—with 99% or 75% mortality of all aquatic stages (eggs, larvae, and pupae) for a single day, respectively.

### Population structure

We investigated the possibility of hidden population structure in our temporal samples using the Bayesian clustering algorithm implemented in the program STRUCTURE version 2.3.4 (Pritchard et al. 2000). Simulations for each value of *K* (1 through 3) were replicated 20 times. For each simulation, a Monte Carlo Markov chain (MCMC) was run for 1 × 10^6^ iterations after a 10 000 step burn-in under a population admixture model assuming independent alleles and without using prior population information. Under the admixture model, each individual draws a fraction of its genome from each of *K* subpopulations. The mostly likely value of *K* was determined based on the ad hoc likelihood measure *L*(*K*) (Pritchard et al. 2000) and visualized using the web program Structure Harvester v0.6.93 (Earl and vonHoldt 2012).

## Results

### Genetic variation

One to two loci were suspected of harboring null alleles for each time point based on the Micro-Checker analyses. However, all the loci were in Hardy–Weinberg equilibrium for each time point, indicating that any null alleles present were at very low frequency and that they pose little, if any, problem for our analyses. Only three of the 936 comparisons showed significant linkage disequilibrium (before Bonferroni correction). Average genetic diversity estimates for the final data set consisting of genotypes for 791 individuals and 13 loci are listed in Table S2. The average *H*_E_ over all loci ranged from 0.61 to 0.63, and the average *A*_R_ ranged from 7.67 to 8.15. A reduction in effective population size due to vector control could conceivably have lowered *A*_R_ through the loss of rare alleles between our time points. However, a one-way anova did not detect any significant differences between temporal samples in either *A*_R_ or *H*_E_ (*A*_R_: *F*_5,77 _= 0.093, *P *>* *0.99; *H*_E_: *F*_5,77 _= 0.47, *P *>* *0.99).

### Approximate Bayesian computation

Of the six demographic scenarios, scenario 2 is by far superior to the virtual exclusion of all others (posterior probability = 0.9926). This scenario models a *N*_*e*_ reduction after each IRS round, followed by an increase in *N*_*e*_ until the next spray round. Posterior probabilities for all other scenarios were <0.0067. Specifically, ABC found no support for scenario 1, which modeled a constant *N*_*e*_. Interestingly, scenario 6, which is similar to scenario 2 but includes a continuous decline for the first 3 months following the first spray round, followed by an increase prior to the second spray round, was not supported. This indicates that the *N*_*e*_ of the population started to recover soon after the June 2009 round. Similarly, scenarios 4 and 5, which were modeled on the assumption of a positive or negative correlation with rainfall, were also unsupported.

**Figure 2:**
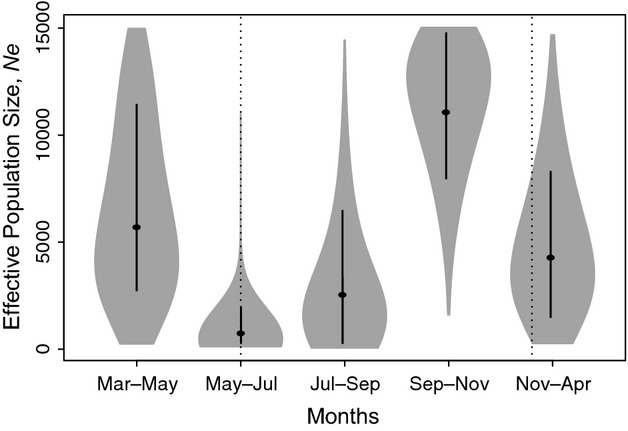
A violin plot for *N*_*e*_ estimates for *Anopheles gambiae* (M) in Punta Europa, Bioko Island, for five intervals during 2009 and early 2010. Horizontal bars indicate the median of the posterior density distribution of *N*_*e*_, and vertical bars indicate the 95% credibility intervals (Cr. I).

Estimates of *N*_*e*_ and their credibility intervals (Cr.I.) for specific times during this study period were derived from the simulations produced under preferred scenario 2 (Table 3). The analysis was not able to obtain more specific estimates of the timing of demographic events than provided by each of the sampling intervals. A visual representation of demographic changes is provided in Fig. 2. In this graph, the dots represent the *N*_*e*_ estimates, and the solid lines represent the 95% Cr.I. The width of the shaded portion indicates the posterior density distribution of the *N*_*e*_ estimates. The dotted lines indicate the approximate timing of two spray rounds. These data show that the *N*_*e*_ of *An. gambiae* in Mongola was approximately 5692 (Cr.I. 2179–12 471) between March and May 2009. This was 5–7 months following a prestudy spray round in October 2008. Between May and July 2009, that is around the June 2009 spray round, *N*_*e*_ drastically declined by approximately 92% to 428 (Cr.I. 230–858). It recovered slightly between July and September 2009 (1782, Cr.I. 176–5477), but then increased dramatically 3–5 months after June 2009 spray round to 12 060 (Cr.I. 8394–14 768). *N*_*e*_ once more drastically declined by 65% to 4204 (Cr.I. 1195–8494), sometime between November 2009 and April 2010, that is around December 2009 spray round.

**Table 3:** Estimates of *N*_*e*_ based on the scenario 2 selected by our ABC analyses. Credibility intervals (95% Cr.I.) are also listed

Interval*	*N*_*e*_	95% Cr.I.
1. March to May 2009	Increased to 5692	2179–12 471
2. May to July 2009	Decreased to 428	230–858
3. July to September 2009	Increased to 1782	176–5477
4. September to November 2009	Increased to 12 060	8394–14 768
5. November 2009 to April 2010	Decreased to 4204	1195–8494

* Column one lists the time interval.

The 95% Cr.I. do not overlap or overlap only slightly for the estimates before and after the three major demographic events: two decreases following spray rounds and the dramatic increase 3–5 months after the first spray round. Credibility intervals in Bayesian analyses represent the uncertainty in the estimate. The *N*_*e*_ estimates around the spray rounds have the narrowest intervals—providing evidence for the greater certainty of population contraction from May 2009 to July 2009 and from November 2009 to April 2010 intervals.

Scenario 2 was also by far the best supported when assuming 12 (posterior probability = 0.979) or 24 (posterior probability = 0.976) generations/year. Additionally, although *N*_*e*_ estimates varied somewhat between these analyses, the results are remarkably robust with respect to this assumption. That is, the overall pattern of demographic changes provided is remarkably similar (Table S3), with dramatic reductions around the time of the spray rounds and equally dramatic population recovery 3–5 months later.

Eight summary statistics that are appropriate for our data set were used to compare the competing demographic scenarios in our analyses. We examined how much each of these statistics contributes to our results by removing one at a time while comparing scenario 2 against either 2nd best-supported scenario (#5) or scenario 1 which represents a constant population size. The expectation is that the removal of a summary statistic with a large influence over the outcome should reduce the posterior probability of the favored scenario. Scenario 2 remained by far the best-supported scenario in all comparisons (Table S6). When comparing scenario 2 vs. the constant *N*_*e*_ scenario, no single summary statistic had a noticeable impact on the outcome, and the posterior probabilities of scenario 2 remained above 0.98. In the comparison between scenario 2 vs scenario 5, one summary statistic, the mean number of alleles, did have a noticeable impact, although scenario 2 was still by far the best supported. Its posterior probability dropped to 0.78 and 0.64, depending on whether the mean number of alleles was removed as a single or pairwise summary statistic, respectively. This indicates that our results are determined by multiple summary statistics rather than a single one.

### Other *N*_*e*_ estimating methods

Three other packages for estimating were also applied to our data: Colony, LDN_*e*_, and MLN_*e*_. Little consistency was evident between them. First, the Colony *N*_*e*_ estimates ranged between 71 and 118 for the six temporal samples with largely overlapping confidence intervals (Table S4). Second, an even less informative result was obtained from the LDN_*e*_ analyses. Although excluding rare alleles from LDN_*e*_ analyses is a recommended practice to reduce bias in *N*_*e*_ estimates (Waples and Do 2010), our point estimates from this method differed substantially depending on whether rare alleles with a frequency <0.02 or <0.005 were excluded (Table S4). When alleles with frequencies <0.02 were removed as recommended by Waples and Do (2010), the *N*_*e*_ estimates of four of the six time points were infinite, while *N*_*e*_ estimates for March and May 2009 were 498 (271–2098) and 464 (276–1241), respectively. When excluding only alleles with a frequency <0.005 from the analyses, *N*_*e*_ estimates ranged from 267 to 1929, but the 95% CI of four of the six time points included ∞. These were the same time points that were infinite with the exclusion at <0.02 frequency, rather than <0.005 frequency. With one exception (May 2009 vs November 2009), these confidence intervals overlapped, often broadly. Finally, Table S5 shows the estimates of *N*_*e*_ using the temporal estimator of Wang and Whitlock (2003) in MLN_*e*_ implemented across sampling intervals of 8–10 generations. Estimates range from 349 (CI 253–500) to 855 (CI 523–1692) and suggest an increase of *N*_*e*_ following the first spray round. In summary, little or no consistency was found between (or within) the results of these other methods of *N*_*e*_ estimation.

### *N*_*e*_ and rainfall

**Figure 3:**
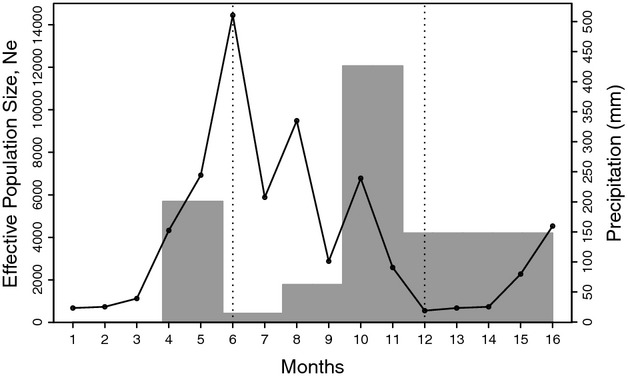
*N*_*e*_ estimates of *Anopheles gambiae* (M) in Punta Europa, Bioko Island (bars), and monthly precipitation amount (line). Actual rainfall data were collected for the period from March to November 2009 in Punta Europa. Monthly averages from the years 2004–2007 were used for the months for which no 2009 data were available. The dashed lines show the approximate timing of the spray rounds in June 2009 and December 2009.

**Figure 4:**
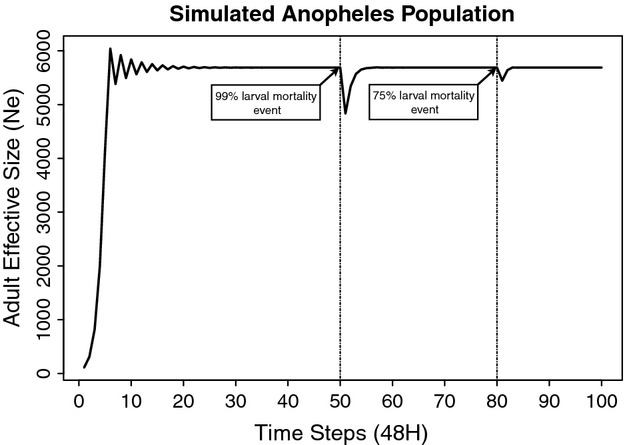
The effect of large egg–larval–pupal mortality on *Anopheles gambiae* effective population size as indicated by our *post hoc* demographic model. Two events were modeled, a 99% and 75% mortality of eggs–larvae–pupae for a single day.

The *N*_*e*_ estimate for the five sampling intervals is represented in Fig. 3 together with the monthly precipitation amount in Punta Europa. *N*_*e*_ is lowest during periods of high precipitation, but *N*_*e*_ increased dramatically before the period of high precipitation ended. The high amount of rainfall recorded in June 2009 is to a large extent due to the extraordinary rainfall on June 22, 2009. To examine the possibility that this single large rainfall event resulted in the observed reduction in *N*_*e*_, we performed a *post hoc* perturbation analysis on a demographic model based on available life table data for *An. gambiae* (Olayemi and Ande 2009). Figure 4 shows the results of the *post hoc* perturbation analyses. With both the 99% and 75% egg–larval–pupal mortality, *N*_*e*_ drops slightly to approximately 4835 and 5444, respectively, and recovers within a few days. This limited impact on *N*_*e*_ of a single large egg–larval–pupal mortality event is perhaps not surprising if one considers that a substantial amount of adult female mosquitoes are reproductively active for a period longer than 10 days (the approximate time of egg to pupal development). This would have allowed many of them to contribute to the next generation, even if all the offspring from a single egg batch were killed. These modeling results clearly demonstrate that the strong *N*_*e*_ reduction indicated by the ABC analysis, which lasted for over a 2-month period, is not due to the extraordinary amount of rainfall on June 22, 2009 in Punta Europa.

### Population structure

Analysis of our data set found no evidence for any population structure within the *An. gambiae* population in Mongola. The STRUCTURE analyses showed that *K* = 1 has the highest posterior probability (Figure S1). Additionally, the probability of assignment under *K* = 2 shows that all individuals within our data set have an equal probability of belonging to either of the two clusters (Figure S2).

## Discussion

No effect of single IRS rounds on genetic variability (*A*_R_ or *H*_E_) is evident in our data. Reductions in population size can reduce *A*_R_ by removing rare alleles, and a campaign against lymphatic filariasis did significantly reduce allelic richness in a *Culex quinquefasciatus* population in Brazil (Cartaxo et al. 2011). However, in populations that have fluctuated multiple times over the course of the last hundred generations or so, few rare alleles remain to be lost, and we would not necessarily expect to see rare allele loss reflected in *A*_R_ during one of these periodical population size reductions. That being said, the mean number of alleles did have some impact on our results, as it was the most influential summary statistic, both as single and pairwise measures, when comparing scenario 2 to the second best scenario. *H*_E_ will be reduced by an increased amount of inbreeding associated with small population size. However, the *An. gambiae* population in Mongola, while greatly reduced, still numbers in several thousands. Thus, inbreeding would increase by an amount too small to measure, the fact that we did not observe a decrease in *H*_E_ is not unexpected.

Our ABC analysis strongly supported a model in which IRS rounds have a big impact on *N*_*e*_, but in which *N*_*e*_ recovers equally dramatically between spray rounds. The effective population size of *An. gambiae s.s*. in the Punta Europa area of Bioko Island fluctuated widely between IRS rounds during 2009 and early 2010. *N*_*e*_ was large in early 2009 and declined by a dramatic 92% around the June 2009 spray round, even as the wet season began. Although we cannot infer the exact timing of the *N*_*e*_ reduction within the May–July 2009 interval, no other event besides the spray round can explain this dramatic event. Interestingly, between 3 and 5 months following the insecticide spraying, the mosquito population had increased by 2818%. Again, the exact timing for this *N*_*e*_ estimate is not known, but assuming a continuing decline in residual efficacy of the carbamate insecticide, combined with the ongoing rainfall, it is reasonable to suspect that *N*_*e*_ was larger toward the end of this interval. No support was found for scenarios based on correlation of *N*_*e*_ with rainfall or constant *N*_*e*_.

It has been shown that the presence of cryptic populations can result in incorrect inferences of population bottlenecks or expansions when reconstructing demographic histories using ABC (Peter et al. 2010). We examined our data for population structure and did not find any. However, we cannot rule out that some temporary and microgeographic structure is present in our study population that we are not able to detect. We do not have any reason to suspect that this is the case, but we do not know what the effect of such subtle population structure on our analyses would be.

There was little consistency evident between the three variance-based *N*_*e*_ estimation methods (Colony, LDN_*e*_, and MLN_*e*_). It is important to note, however, that variance-based methods are most precise for smaller populations and may not be appropriate for our data set. In contrast, ABC is a highly robust method due to its ability to use the variances from multiple summary statistics, instead of single variance components used by other *N*_*e*_ approaches. This allows more information from the observed data set to be used to infer complex demographic histories (Cornuet et al. 2008, 2010; Luikart et al. 2010). Additionally, ABC is not constrained by many assumptions found in non-Bayesian *N*_*e*_ methods, which inherently makes it less biased due to assumption violations (Luikart et al. 2010; Tallmon et al. 2010). Lastly, perhaps the greatest strength of ABC is that it allows the comparisons of alternative complex scenarios. Certainly in our study, the ABC approach was able to provide a remarkably detailed picture of fluctuations in *N*_*e*_ of a large population, even using samples collected only about three generations apart.

It is well known that rainfall patterns affect fluctuations in mosquito effective population size (Taylor et al. 1993; Simard et al. 2000; Midega et al. 2010), but in our study, this effect was overshadowed by the impact of insecticide spraying and the apparent limitation on the residual efficacy of the carbamate insecticide used in the IRS program. That being said, it is likely that precipitation has some effect on *N*_*e*_ and that IRS may have a larger or smaller impact in different seasons. A longer study over more than two spray rounds and including multiple years would be needed to shed light on this.

A heavy downpour deposited 177 mm of rain on Punta Europa on June 22, 2009, only 10 days following the first spray round. This could have temporarily reduced mosquito *N*_*e*_ as eggs, larvae, and pupae might have been washed from their habitat. It is certainly conceivable that flooding would remove *An. gambiae* larvae from the small temporary breeding pools, and one study has shown that larval mortality can increase during nights with rainfall (Paaijmans et al. 2007). However, the opposite can also occur because very large numbers of *An. gambiae s.l*. have been reported in Burkina Faso after a period of heavy rains and flooding (Costantini et al. 1996). Our *post hoc* demographic modeling indicates that the single night of very large rainfall is unlikely to be the cause of the dramatic and prolonged reduction in *N*_*e*_. Even under an extreme scenario that assumes 99% mortality of all aquatic stages on the day of the deluge, the predicted *N*_*e*_ reduction (16%) was much smaller and much more temporary than was observed in our study.

**Figure 5:**
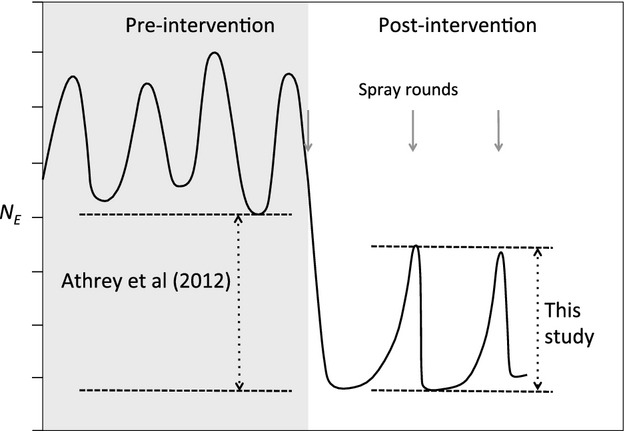
A visualization of the fluctuations in *N*_*e*_ in *Anopheles gambiae* prior and post-IRS interventions based on the results from Athrey et al. 2012b; and this study. Before the IRS campaign, seasonal fluctuations existed within the populations. After the IRS campaign started, *N*_*e*_ was reduced but continues to fluctuate, now largely due to the timing of IRS rounds.

Previously, we demonstrated a 76%–85% reduction in the *N*_*e*_ of three malaria vectors coincidental with the start of IRS programs (Athrey et al. 2012b). Those observations can be combined with the large fluctuations found in this study to construct a model for understanding the dynamics of *An. gambiae* populations before and after the start of IRS-based vector control (Fig. 5). According to this model, the estimated pre-intervention *N*_*e*_ in Mongola approximates the lowest value of *N*_*e*_ (15 700) during the seasonal fluctuations before the start of the IRS program (Athrey et al. 2012b). After the initiation of IRS, fluctuations in *N*_*e*_ are predominantly determined by the timing of spray rounds, and low *N*_*e*_ values during these fluctuations are the postintervention *N*_*e*_ of 3290 for 2004 reported by Athrey et al. (2012b), as well as the *N*_*e*_ of 428 for May–July 2009 and 4204 for November 2009–April 2010 reported in this study.

Our observation that the *An. gambiae* population in Punta Europa rebounded dramatically between 3 and 5 months after spraying of carbamate insecticide has important implications for vector control. Bradley et al. (2012) have recently shown that the mortality of mosquitoes in WHO standard cone bioassays performed on walls sprayed with carbamate on Bioko Island declined from 100% immediately after a spray round to 73% (2010)–81% (2011) after 4 months, and 69% (2010)–78% (2011) after 5 months. If the residual efficacy of the carbamate insecticide was similar in 2009–2010 and 2011, only an approximate 20%–30% drop in mosquito mortality resulted in almost complete rebounding of mosquito populations. This suggests that this modest reduction in mortality has a huge impact on the mosquito population's ability to recover or that the WHO assay does not represent well the level of mortality in the *An. gambiae* population. This could be because the length of exposure to sprayed walls during the bioassays may not equal actual exposure length of wild *An. gambiae* mosquitoes. Bradley et al. (2012) also showed an increase in infection prevalence in the human population from 21% to 28% between 4 and 5 months and concluded that IRS rounds start to lose effectiveness after 4 months. This conclusion is underscored dramatically by the results of this study, which show a drastic rebounding of the *An. gambiae* population. Therefore, although IRS campaigns have proven effective, they should not be expected to have a lasting impact on *An. gambiae* populations, unless of course populations are eliminated. Similarly, even a temporary interruption of IRS activities will likely result in a rapid resurgence in mosquito population size and presumably malaria transmission.

Typically, IRS rounds are spaced 6–12 months apart, depending on the seasonality of the vector and available resources. On Bioko Island, spray rounds have been conducted twice yearly since 2005. Our results show that in regions where transmission occurs throughout the year, biannual spray rounds are likely to have a suboptimal effect on transmission. The large resurgence observed in *An. gambiae N*_*e*_ only 3–5 months after a spray round indicates that spraying should be conducted at most 4 months apart to have a optimal impact on mosquito populations and hence malaria transmission. During 2012, the BIMCP has increased the rate of spray rounds to three per year in the Punta Europa area in an effort to reduce malaria prevalence in this area of continued high transmission. Rowland et al. (2000) showed that the residual efficacy of the often used pyrethroid alpha-cypermethrin starts to decline 4–4.5 months after spraying and is around 70% 5 months after spraying. This suggests that more frequent spraying compared with what is currently practiced may provide a great benefit to IRS programs using other insecticides as well.

The success of IRS depends on a sufficiently high level of endophily in vector populations. While *An. gambiae* has generally been considered a largely indoor feeding and resting species, it was recently shown that vector populations can shift to more outdoor feeding behavior following indoor-based vector control (Russell et al. 2011). This is also the case in the Punta Europa area of Bioko Island, where human landing catches conducted in 2007–2009 collected more mosquitoes outdoors than indoors, whereas none were reported before (Reddy et al. 2011). This is major source of concern, not only for the ability of IRS and LLINs/ITNs to reduce mosquito populations, but also because people are not protected from infective mosquitoes when outdoors during the evening hours. Although some cause for concern remains, our results do show that despite a large amount of outdoor feeding, IRS remains capable of having a large impact on mosquito population sizes.

An important question related to our study is how estimates of *N*_*e*_ compared with the census size (*N*_*c*_). Comparisons between direct methods such as MRR and estimates of *N*_*e*_ are available for at least one malaria vector population. Touré et al. (1998) used MRR to estimate that the *An. arabiensis* population size in Banambani, Mali, is between 9073 and 36 249 during the wet season. This is approximately 4- to 6-fold the *N*_*e*_ of the same population, which was estimated between 2230 and 5892 (Taylor et al. 1993). Although natural populations rarely behave like the ideal population and *N*_*C*_ therefore seldom equals *N*_*e*_, the difference between these estimates is undoubtedly exaggerated by the fact that Banambani experiences a severe dry season with 90% of the rain falling between June and September (Taylor et al. 1993). The *N*_*e*_ estimate is likely to be closer to the minimum size during the dry season, whereas the MRR estimated the population size after the dry season. Therefore, in our study, *N*_*e*_ may be somewhat closer to *N*_*c*_ than reported by Taylor et al. (1993) and Touré et al. (1998).

The samples used in our study were collected as part of the vector monitoring component of the BIMCP. Mosquitoes were collected during biweekly light trap collections (LTCs) between February 16, 2009 and November 30, 2009, as well as during human landing catches (HLCs) five times during 2009 (Overgaard et al. 2012). A comparison of these LTCs, HLCs, and our *N*_*e*_ estimates underscores the difficulty of estimating mosquito abundance from collection methods. The light trap collections indicated a high abundance of mosquitoes between April and the first half of June, dropping off to very low numbers between July and August. Starting in August 2009, the number of *An. gambiae* collected increased slightly but remained low. So, the drastic increase in *N*_*e*_ in October was not represented well in the LTCs. Conversely, the number of *An. gambiae* collecting during HLCs did double during September 2009 collections, but no drop in HLC numbers was observed before and after the first spray round. Therefore, consistency between the two collection methods was low.

## Conclusion

The most important implication of our study is that IRS as currently practiced is not meeting its full potential for reducing malaria transmission. Although insecticide spray rounds had a dramatic effect on the effective population size of the malaria vector *An. gambiae* in an area highly endemic for malaria, this effect was considerably shorter than the 6 months separating spray rounds. As soon as the residual efficacy of the insecticide began to decline, the mosquito population increased by 2818% and approached precontrol *N*_*e*_. From this study and considering the limitations on the residual efficacy of other insecticides (Rowland et al. 2000; N'Guessan et al. 2007), then follows that increasing the frequency of spray rounds from what is typically practiced in IRS programs is likely to dramatically increase their impact on mosquito populations and malaria transmission, at least in areas of very high transmission. Finally, our study also has implications for evolutionary biologists, as our comparison with other methods of *N*_*e*_ estimation highlights the ability of ABC to detect patterns of multiple population contraction/expansion events across only approximately 30 generations in a large population. ABC and similar coalescent approaches have opened up a variety of options to study the population dynamics of medically important vectors and pathogens, whose demographic and evolutionary histories are poorly understood, and a better characterization of which will greatly enhance our ability to fight infectious diseases around the world.

## Data archiving

The data used in this article has been deposited in the Dryad repository: doi:10.5061/dryad.35rs4.

## Supporting Information

**Table S1.** A description of demographic models tested using approximate Bayesian computation on multiple time-point samples from *A. gambiae* (M-form) from Mongola, Bioko Island. **Table S2.** Average heterozygosity (H_E_), allelic richness (A_R_) and their standard errors (SE) for the *An. gambiae* population in Mongola for six temporal samples. **Table S3.** Estimates of N_e_ based on scenario 2 when using 12 and 24 generations/year. Credibility intervals (95% Cr.I.) are also listed in parentheses. **Table S4.** Results from the two point estimators of effective population size (N_e_), with 95% confidence intervals provided in parenthesis. **Table S5.** Estimates from the maximum likelihood temporal estimator MLNe. N_e_ estimates and corresponding 95% confidence intervals are presented. **Table S6.** Results from sensitivity analyses examining the effect of each summary statistic on ABC model choice. **Figure S1.** Results of STRUCTURE analysis indicating likelihood (LnP(K)) scores of K populations (1–3) and variance per K value. **Figure S2.** Bayesian assignment probabilities for two putative populations (K = 2). The optimal number of K equals 1. K = 2 is shown to demonstrate the complete lack of structure within the populations.
