# Supplementary material for: Large fluctuations in the effective population size of the malaria mosquito Anopheles gambiae s.s. during vector control cycle
Source: Evol Appl. 2013 Aug 7;6(8):1171–83. doi: 10.1111/eva.12094 (PMC3901547; doi:10.1111/eva.12094)

Table S1 - A description of demographic models tested using Approximate Bayesian Computation on multiple time-point samples from *An. gambiae* (M-form) from Mongola, Bioko Island.

Scenario	Parameter	Description	Prior[1]			Steps	Conditions Enforced
			12 gen	18 gen	24 gen		
1<br>N_e is constant	NpC	present population size	100:15000			100	
	t1	time at which population has size of NhistC	14-64	20-70	27-77	1	
	NhistC	historical population size prior to March 2009	100:15000			100	
2<br>N_e is reduced by IRS rounds, but starts to recover soon after	NpVC1	present population size	100:15000			100	
	NhistVC1	historical population size prior to March 2009	100:15000			100	NhistVC1<NmVC1i1
	NmVC1d1	population size after decrease due to effects of 1st spray round	100:15000			100	NmVC1d1<NmVC1i2
	NmVC1d2	population size after decrease due to effects of 2nd spray round	100:15000			100	NmVC1d2≥NpVC1
	NmVC1i1	population size after increase between March and May 2009	100:15000			100	NmVC1i1>NmVC1d1
	NmVC1i2	population size after increase between July and September 2009	100:15000			100	NmVC1i2<NmVC1i3
	NmVC1i3	population size after increase between September and November 2009	100:15000			100	NmVC1i3>NmVC1d2
	t1	time at which population has a size of NhistVC1	14-64	20-70	27-77	1	
	t2	time at which NhistVC1 changes to NmVC1i1	12-13	17-19	23-25	1	
	t3	time at which NmVCi1 changed to NmVC1d1	10-11	14-16	19-21	1	
	t4	time at which NmVC1d1 changed to NmVC1i2	8-9	11-13	15-17	1	
	t5	time at which NmVC1i2 changed to NmVC1i3	6-7	8-10	11-13	1	

	t6	time at which NmVC1i3 changed to NmVC1d2	1-5	1-6	1-9	1	
3 N_e is reduced by each subsequent IRS round and does not recover between rounds.	NpVC2	present population size		100:15000		100	
	NhistVC2	historical population size prior to March 2009		100:15000		100	NhistVC2>NmVC2d1
	NmVC2d1	population size after decrease due to effects of 1st spray round		100:15000		100	NmVCd1>NmVC2d2
	NmVC2d2	population size after decrease due to effects of 2nd spray round		100:15000		100	NmVC2d2≥NpVC2
	t1	time of NhistVC2	14-64	20-70	27-77	1	
	t3	time at which NhistVC2 changed to NmVC2d1	10-11	14-16	19-21	1	
	t6	time at which NmVC2d1 changed to NmVC2d2	1-5	1-6	1-9	1	
4 N_e fluctuates based on a positive correlation with rainfall.	NpR1	present population size		100:15000		100	
	NhistR1	historical population size prior to March 2009		100:15000		100	NhistR1<NmR1i1
	NmR1d1	population size after decrease due to lower rainfall amounts between September and November 2009		100:15000		100	NmR1d1>NmR1d2
	NmR1d2	population size after decrease due to lower rainfall amounts between November 2009 and April 2010		100:15000		100	NmR1d2≥NpR1
	NmR1i1	population size after increase due to higher rainfall amounts between March and May 2009		100:15000		100	NmR1i1<NmR1i2
	NmR1i2	population size after increase due to higher rainfall amounts between May And July 2009		100:15000		100	NmR1i2>NmR1d1
	t1	time at which population has size of NhistR1	14-64	20-70	27-77	1	
	t2	time at which NhistR1 changed to NmR1i1	12-13	17-19	23-25	1	
	t3	time at which NmR1i1 changed to NmR1i2	10-11	14-16	19-21	1	
	t5	time at which NmR1i2 changed to NmR1d1	6-7	8-10	11-13	1	
	t6	time at which NmR1d1 changed to NmR1d2	1-5	1-6	1-9	1	

5<br>N_e fluctuates based on a negative correlation with rainfall.	NpR2	present population size		100:15000		100	
	NhistR2	historical population size prior to March 2009		100:15000		100	NhistR2>NmR2d1
	NmR2d1	population size after decrease due to higher rainfall amounts between March and May 2009		100:15000		100	NmR2d1>NmR2d2
	NmR2d2	population size after decrease due to higher rainfall amounts between May And July 2009		100:15000		100	NmR2d2<NmR2i1
	NmR2i1	population size after increase due to lower rainfall amounts between September and November 2009		100:15000		100	NmR2i1<NmR2i2
	NmR2i2	population size after increase due to lower rainfall amounts between November 2009 and April 2010		100:15000		100	NmR2i2≤NpR2
	t1	time at which population has size of NhistR2	14-64	20-70	27-77	1	
	t2	time at which NhistR2 changed to NmR2d1	12-13	17-19	23-25	1	
	t3	time at which NmR2d1 changed to NmR2d2	10-11	14-16	19-21	1	
	t5	time at which NmR2d2 changed to NmR2i1	6-7	8-10	11-13	1	
	t6	time at which NmR2i1 changed to NmR2i2	1-5	1-6	1-9	1	
6<br>N_e is reduced by IRS rounds and declines for 3 months before recovering by month 4.	NpVC3	present population size		100:15000		100	
	NhistVC3	historical population size prior to March 2009		100:15000		100	NhistVC3>NmVC3d1
	NmVC3d1	population size after decrease due to effects of 1st spray round		100:15000		100	NmVC3d1<NmVC3i1
	NmVC3d2	population size after decrease due to effects of 2nd spray round		100:15000		100	NmVC3d2≥NpVC3
	NmVC3i1	population size after increase between September and November 2009		100:15000		100	NmVC3i1>NmVC3d2

	t1	time at which population size has a size of NhistVC3	14-64	20-70	27-77	1	
	t3	time at which NhistVC3 changed to NmVC3d1	10-11	14-16	19-21	1	
	t5	time at which NmVC3d1 changed to NmVC3i1	6-7	8-10	11-13	1	
	t6	time at which NmVC3i1 changed to NmVC3d2	1-5	1-6	1-9	1	

[1] Priors for times in the demographic models are based on generation times of 12, 18 or 24 for *An. gambiae*. All priors are based on a uniform distribution.

Table S2. Average heterozygosity (*H_E*), allelic richness (*A_R*) and their standard errors (S.E.) for the *An. gambiae* population in Mongola for six temporal samples.

**Sampling Time Points**	***H_E***	**S.E.**	***A_R***	**S.E.**
March 2009	0.632	0.043	7.788	0.442
May 2009	0.619	0.051	8.064	0.689
July 2009	0.638	0.043	8.151	0.596
September 2009	0.620	0.044	8.09	0.618
November 2009	0.624	0.050	7.894	0.778
April 2010	0.610	0.044	7.671	0.567

Table S3. Estimates of N_e based on scenario 2 when using 12 and 24 generations/year. Credibility intervals (95% Cr.I.) are also listed in parentheses.

**Interval**[1]	N_e	
	**12 generations/year**	**24 generations/year**
1 - March to May 2009	increased to 6,808 (2,725-13,500)	increased to 7,480 (3,294-14,379)
2 - May to July 2009	decreased to 344 (216-805)	decreased to 1,190 (480-2,452)
3 - July to Sept 2009	increased to 2,016 (158-6,293)	increased to 2,760 (346-6,555)
4 - Sept to Nov 2009	increased to 10,020 (5,174-14,678)	increased to 11,100 (6,475 - 14,788)
5 - Nov 2009 to April 2010	decreased to 2,856 (748-6,906)	decreased to 4,490 (1,221-7,687)

[1] The time intervals between the temporal samples.

Table S4. Results from the two point estimators of effective population size (N_e), with 95% confidence intervals provided in parenthesis.

Year	Sample Point	Sample Size	Ne *(Colony)*	Ne (*LDNe*) 0.02	Ne (*LDNe*) 0.005
2009	March	125	96 (72-129)	498 (271 - 2098)	953 (379 - ∞)
	May	142	111 (84-148)	464 (276 - 1241)	180 (110 - 391)
	July	137	98 (73-128)	∞ (1733 - ∞)	267 (125 - 3103)
	September	147	118 (90-158)	∞ (2409 - ∞)	687 (254 - ∞)
	November	146	105 (79-139)	∞ (943 - ∞)	1929 (554 - ∞)
2010	April	94	71 (51-103)	∞ (865 - ∞)	653 (233 - ∞)

Table S5. Estimates from the maximum likelihood temporal estimator *MLN_e*. N_e estimates and corresponding 95% confidence intervals are presented.

Sampling Interval	Sample Time Points	*MLNe*	95% C.I.
**8 Generations**	March-July 2009	349	253-500
	July-November 2009	642	422-1086
	May-September 20009	855	523-1692
**10 Generations**	Nov 2009 - Apr 2010	667	440-1121

Table S6. Results from sensitivity analysis that examines the effect of each summary statistic on ABC model choice.

Summary Statistic Removed	Posterior Probabilities of Competing Scenarios			
	Vector Control vs Rainfall		Vector Control vs Constant	
	Vector Control	Rainfall	Vector Control	Constant
**Single Summary Statistic**				
Mean number of alleles	0.7776	0.2224	0.9965	0.0035
Mean genic diversity	0.9979	0.0021	1.0000	0.0000
Mean size variance	0.9973	0.0027	1.0000	0.0000
Mean Garza-Williamson's M	0.9995	0.0005	1.0000	0.0000
**Pairwise Summary Statistic**				
Mean number of alleles	0.6435	0.3565	0.9918	0.0082
Mean genic diversity	0.9968	0.0032	0.9963	0.0037
Mean size variance	0.9971	0.0029	1.0000	0.0000
Fst	1.0000	0.0000	0.9825	0.0175

Figure S1. Results of STRUCTURE analyses indicating likelihood (LnP(K)) scores of *K* populations (1-3) and variance per *K* value.

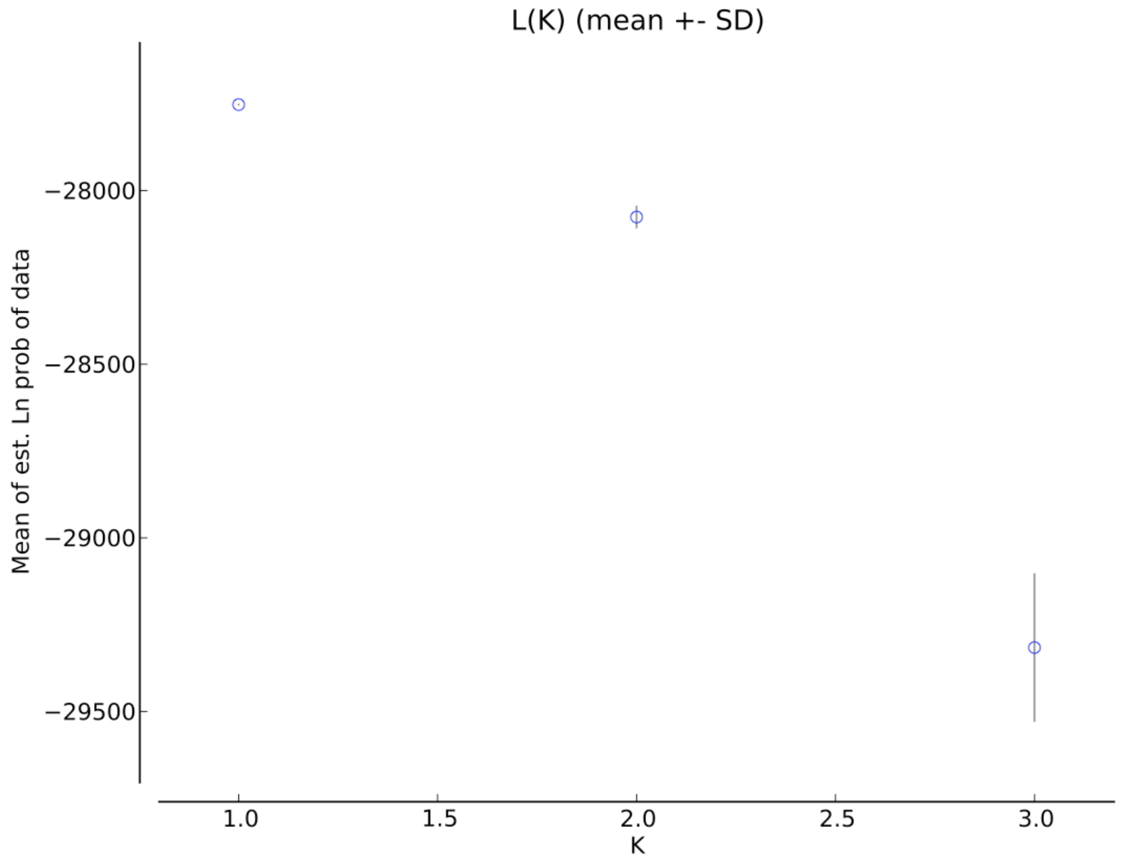

Figure S2. Bayesian assignment probabilities for two putative populations (*K*=2). The optimal number of K equals 1. K=2 is shown to demonstrate the complete lack of structure within the populations.

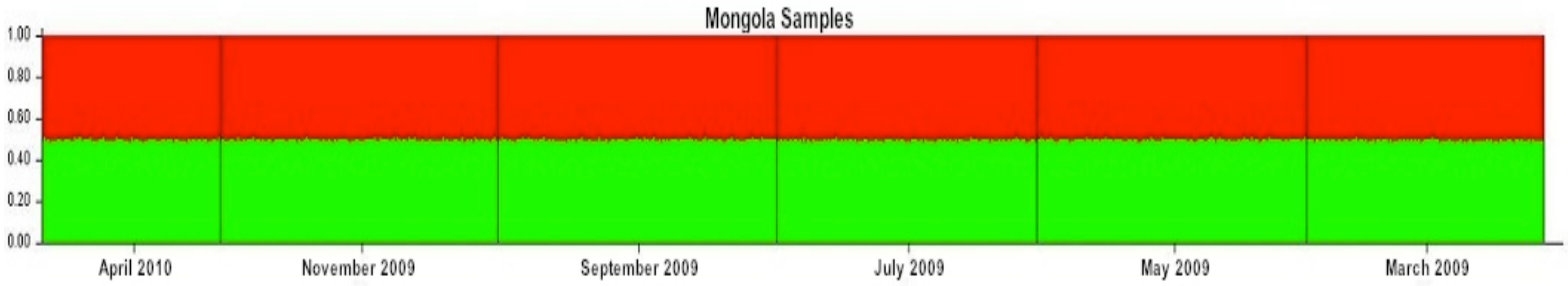

Supplement: Supplementary file 1 — Table S1. A description of demographic models tested using approximate Bayesian computation on multiple time-point samples from A. gambiae (M-form) from Mongola, Bioko Island. Table S2. Average heterozygosity (HE), allelic richness (AR) and their standard errors (SE) for the An. gambiae population in Mongola for six temporal samples. Table S3. Estimates of Ne based on scenario 2 when using 12 and 24 generations/year. Credibility intervals (95% Cr.I.) are also listed in parentheses. Table S4. Results from the two point estimators of effective population size (Ne), with 95% confidence intervals provided in parenthesis. Table S5. Estimates from the maximum likelihood temporal estimator MLNe. Ne estimates and corresponding 95% confidence intervals are presented. Table S6. Results from sensitivity analyses examining the effect of each summary statistic on ABC model choice. Figure S1. Results of STRUCTURE analysis indicating likelihood (LnP(K)) scores of K populations (1–3) and variance per K value. Figure S2. Bayesian assignment probabilities for two putative populations (K = 2). The optimal number of K equals 1. K = 2 is shown to demonstrate the complete lack of structure within the populations. [file eva0006-1171-sd1.pdf]
